# Yunnan Baiyao (YNBY): pharmacological mechanisms, therapeutic applications, and clinical evidence

**DOI:** 10.3389/fphar.2025.1589035

**Published:** 2025-07-09

**Authors:** Hongjing Qian, Wanli Zhang, Mingzhu Zhang, Sijia Ma, Manhong Zhen, Hongbing He, Xiaobin Ren

**Affiliations:** ^1^ Yunnan Key Laboratory of Stomatology, School of Stomatology, Kunming Medical University, Kunming, China; ^2^ Department of Periodontology, Kunming Medical University School and Hospital of Stomatology, Kunming Medical University, Kunming, China

**Keywords:** Yunnan Baiyao, botanical drug, clinical application, hemostatic efficacy, traditional Chinese medicine

## Abstract

Yunnan Baiyao (YNBY) is a traditional Chinese medicine widely used in hemostasis, anti-inflammation, antibacterial, analgesia and wound healing. This article reviews the research progress on the pharmacological mechanism, clinical application and safety of YNBY in the past two decades. Through systematic analysis of clinical trials, animal experiments and mechanistic studies, YNBY has shown significant effects in reducing surgical bleeding (such as reducing blood loss in orthognathic surgery by 21.4%), accelerating wound healing (such as increasing the healing rate of diabetic ulcers by 37%), regulating inflammation (such as inhibiting the expression of IL-6/CRP in arthritis), and inhibiting bacterial virulence (such as destroying *Staphylococcus aureus* biofilm). Mechanistic studies have shown that YNBY acts through multiple pathways, including platelet activation, arachidonic acid metabolism regulation and autophagy regulation. However, existing studies have limitations such as inconsistent trial design and insufficient long-term safety data. This study provides a theoretical basis for optimizing the clinical application of YNBY, and points out future research directions, including the identification of active ingredients, the development of targeted delivery systems, and the development of large-scale randomized controlled trials.

## 1 Introduction

Yunnan Baiyao (YNBY) is a traditional Chinese medicine formula discovered by Qu Huanzhang, a well-known Yunnan physician, in 1902. Historically, it has been widely used for wound healing, trauma-related bleeding, and inflammation, and gained recognition during the Vietnam War for its efficacy and accessibility. Ethnographic records indicate that YNBY was traditionally used by the Yi and Bai ethnic groups, who utilized YNBY powder topically for wound repair or orally to resolve internal bleeding. Over the past century, YNBY has been developed into a variety of formulations, including capsules, tinctures, and toothpastes, while maintaining its status as a protected state secret formula (“National Chinese Medicine Secret Varieties”) (Li et al., 2020).

Although the ingredients of some YNBY formulations are not disclosed, they have been characterized by pharmacological and patent analyses. Core components include Panax notoginseng (Burkill) F.H. Chen (Araliaceae)-Sanqi (Pharmacopoeia of China 2020)—a major component rich in saponins (R1, Rg1, and Rb1) that has hemostatic, anti-inflammatory, and microcirculatory effects; Aconitum carmichaelii Debeaux (Ranunculaceae)-Chuanwu (Monograph WHO 2007), whose toxicity is reduced by 90% after processing, and possesses analgesic and anti-inflammatory effects; Dryobalanops aromatica C.F. Gaertn. (Dipterocarpaceae)-Borneol (ISO 856:2006), which promotes transdermal absorption of the drug (1%–3% in topical preparations) and exhibits antimicrobial activity; Paris polyphylla Sm. (Melanthiaceae)-Chonglou (EP 10.0), which contains steroidal saponins with hemostatic and antitumor effects; Moschus moschiferus Linnaeus (Moschidae)-Shexiang (CITES Appendix II), which counterbalances the toxicity of the formulation and stabilizes bioactive compounds; Glycyrrhiza uralensis Fisch. (Fabaceae)-Gancao (USP 43); and Angelica dahurica (Hoffm.) Benth. and Hook. f. (Apiaceae)-Baizhi (JP XVIII) (Yao et al., 2021; Li Y. J. et al., 2024).

Quality control was performed in accordance with WHO guidelines, and certified through the Royal Botanic Gardens (Kew) Medicinal Plant Name Service (Section 7). The key production processes for saponins, such as ethanol extraction and supercritical CO_2_ sterilization, ensure the retention of heat-sensitive components (patent CN102526046B, CN104922706A). Modern formulations, such as YNBY toothpaste, incorporate nontraditional additives (such as menthol and tranexamic acid), while maintaining core therapeutic principles.

Recent studies have expanded the use of YNBY to cancer therapy and bone protection, mediated by mechanisms such as osteoclast inhibition and osteoblast activation. This review systematically searched five databases, including PubMed and Web of Science, from January 2000 to March 2025, using the key words “yunnanbaiyao” and “YNBY”, combined with pharmacological action words such as hemostasis and anti-inflammation, and research type words such as clinical trial. A total of 87 studies that met the criteria (original studies, including clear dose and quantitative results) were included, as well as case reports, while repeated publications and other articles that did not meet the requirements were excluded. The search process was completed independently by two investigators, and disagreements were resolved by discussion. The pharmacological effects were classified into seven domains: (1) hemostasis, (2) wound healing, (3) anti-inflammation, (4) antibacterial activity, (5) analgesia, (6) anti-tumor activity, (7) and bone protection (Yu et al., 2022; Zhang et al., 2024). In addition, the clinical application, safety profile, and challenges of YNBY were further analyzed, aiming to provide a theoretical basis for promoting research on YNBY and expanding its therapeutic applications.

## 2 Pharmacological effects of YNBY and its mechanism of action

### 2.1 Hemostatic efficacy

The hemostatic efficacy of YNBY has been demonstrated through both clinical and preclinical studies, although important gaps remain in understanding its mechanisms and long-term effects (Tang et al., 2009). In a pivotal clinical trial using a double-blind design, 87 patients undergoing bimaxillary orthognathic surgery who received YNBY capsules preoperatively (dose: 500 mg 3 times daily for 3 days) experienced significantly reduced intraoperative blood loss (330.5 ± 134.4 mL) compared with those who received placebo (420.3 ± 175.9 mL), with no adverse events reported. However, while this study established the short-term safety and efficacy of YNBY, it did not evaluate postoperative recovery or long-term outcomes.

Mechanistic studies have yielded mixed results. A canine model was intravenously injected with YNBY (2 mg/kg) and coagulation function was assessed by thrombelastography (TEG). Normal saline was used as a negative control and tranexamic acid (10 mg/kg) was used as a positive control. YNBY was found to enhance clot strength on TEG while reducing fibrinolysis, although the small sample size (n = 18) and lack of placebo control limit interpretation (Tansey et al., 2018). Conversely, a crossover study in horses found no significant effects on platelet activation or von Willebrand factor function, suggesting potential species-specific responses or dose-dependent effects (Ness et al., 2017).

YNBY has also shown high efficacy in improving blood circulation and enhancing bone healing (Ren et al., 2020). Recent studies have also examined YNBY’s effects on blood circulation and coagulation. One study analyzed how YNBY influences small- and medium-molecule metabolites in rat urine, using untargeted metabolomics with UPLC-G2Si-HDMS technology. Researchers used an epinephrine hydrochloride-induced blood stasis model in rats and identified 28 urinary biomarkers through metabolomic analysis combined with multivariate statistics. The results showed improved blood flow, coagulation function, and pathology in treated rats, suggesting that YNBY exhibits efficacy in treating blood stasis syndrome. The metabolomics strategy provides opportunities for an in-depth understanding of the mechanism of action of YNBY. Studies have found that YNBY may exert its effect of promoting blood circulation and eliminating blood stasis through effects on multiple metabolic pathways. This research method utilizes UPLC-G2Si-HDMS technology combined with metabolomics analysis, which provides a new technological pathway for the study of traditional Chinese medicine. Furthermore, the results provide a scientific basis for analyzing the mechanism of action of YNBY, especially in blood circulation and blood stasis.

The study involved a wide range of metabolic pathways and provided a comprehensive perspective for understanding the multi-targeted effects of YNBY. However, the study mainly focused on animal models, and further clinical studies are needed to verify whether the results can be directly generalized to humans. Although additional biomarkers and metabolic pathways are mentioned in the article, the specific molecular mechanisms and pathways of action have not been fully elucidated, and further studies are needed. However, the multi-targeted mechanism of action of YNBY suggests the need to analyze multiple aspects of the effects of a drug, including at molecular, cellular, and overall levels. In conclusion, metabolomics technology provides valuable insights into the metabolic targets of YNBY in activating blood circulation and alleviating blood stasis, offering an opportunity for more in-depth understanding of its pharmacological effects (Zhang et al., 2022) (Table 1).

**TABLE 1:** Evidence for Yunnan Baiyao’s hemostatic effects: Clinical and preclinical studies.

Study type	Model/Design	Dose and administration	Key findings	Mechanistic insights	Limitations	Ref.
Clinical (Level Ib RCT)	87 patients undergoing bimaxillary orthognathicsurgery	500 mg capsules, 3×/day for 3 dayspre-op	↓Intraoperative blood loss by 21.4% (330.5 ± 134.4 mL vs. placebo 420.3 ± 175.9 mL, *p* < 0.05)	Enhanced fibrin clot stability; no effect on plateletcount	No long-term follow-up; single-centerdesign	Tang et al. (2009)
Preclinical (Canine)	Healthy dogs (n = 18)	2 mg/kg IV (vs. tranexamic acid 10 mg/kg)	↑ Clot strength (TEG: MA +15%, *p* < 0.01); ↓fibrinolysis (LY30%–40%)	GPIIb/IIIa activation confirmed by flow cytometry	Small sample size; no placebocontrol	Tansey et al. (2018)
Preclinical (Equine)	Healthy horses (crossover)	15 mg/kg oral cream, q12 h × 3 days	No significant change in vWF or platelet aggregation (*p* > 0.05)	Species-specific responsesuspected	Lack of bleeding model	Ness et al. (2017)
Metabolomics (Rat)	Epinephrine-induced bloodstasis	180 mg/kg/day oral ×15 days	↓ Plasma viscosity by 30%; ↑ coagulation factors (FII,FVII)	ALOX15/EDN1 pathway modulation (arachidonic acidmetabolism)	Animal model only; human relevanceunclear	Zhang et al. (2022)

While YNBY demonstrates promising hemostatic effects, current studies exhibit notable limitations. Clinical trials, such as the orthognathic surgery study (Tang et al., 2009), have focused on short-term outcomes without assessing long-term safety or postoperative complications. Preclinical models, though informative, often lack placebo controls (Ness et al., 2017) or sufficient sample sizes (Frederick et al., 2017; Tansey et al., 2018), reducing translational reliability. Mechanistically, while YNBY’s impact on fibrinolytic activity and platelet activation is well-documented, its interactions with specific pathways (e.g., thrombin generation, vascular endothelial growth factor) remain poorly understood. For instance, metabolomic associations with ALOX15 (Zhang et al., 2022) suggest a role in arachidonic acid metabolism; however, direct evidence is lacking. Addressing these gaps through longitudinal clinical monitoring and molecular pathway analyses will strengthen the rationale for YNBY’s hemostatic applications.

### 2.2 Wound healing

Beyond its hemostatic effects, YNBY has demonstrated significant wound-healing potential when administered through innovative delivery systems and as part of combination therapies. One study constructed a microneedle patch loaded with YNBY, which was applied to a full-thickness cutaneous defect model to evaluate the ability of the microneedle patch to promote skin wound healing *in vivo*. The effect of (BY + EGF) @MNs on wound healing was observed by creating a circular full-thickness skin defect on the back of rats and applying different treatments. The study showed that (BY + EGF)@MNs significantly promoted hemostasis, neovascularization, fibroblast density, and collagen deposition. This study combined several disciplines such as nanobiotechnology, drug delivery systems, and biomaterials science, demonstrating the advantages of multidisciplinary cross-collaboration in exploring the potential clinical applications of YNBY, especially in trauma care and emergency medical response. However, this study has some shortcomings: although the article provides short-term experimental results, there is insufficient data on the long-term effects, side effects, and safety of the microneedle patches. Further, the study may not have adequately considered differences in response to microneedle patches in different individuals (e.g., based on age, sex, and health status). In addition, although the study compared the effects of different treatment groups, it did not conduct a comparison of the efficacy of the microneedle patches against that of existing hemostatic and wound-healing products. This may limit a comprehensive assessment of the performance of microneedle patches. Overall, although this article provides a valuable contribution, there are still areas where further research is warranted (Yang et al., 2023).

The wound-healing efficacy of YNBY appears particularly pronounced in diabetic models. In a comprehensive evaluation study of the bioactive glass-YNBY combination in streptozotocin-induced diabetic rats, a 5% YNBY-bioactive glass complex was applied topically (once daily) and silver sulfadiazine was used as control. The wound closure rate was quantified by digital photography and ImageJ software. The healing rate of the treatment group was 92% at 21 days, which was significantly higher than that of the control group (78%). The 5% YNBY formula achieved a higher rate of wound closure through enhanced fibroblast proliferation, granulation tissue formation, and angiogenesis compared with the other treatment groups. These findings suggest a synergistic interaction between YNBY’s bioactive components and the ionic microenvironment created by bioactive glass materials, although the precise molecular mechanisms remain to be elucidated (Mao et al., 2014).

Further mechanistic insights come from studies examining YNBY’s anti-inflammatory properties. Comparative research with Chuanwu (Aconitum Carmichaelii) and Hanxia (Pinellia ternata) extracts has demonstrated YNBY’s ability to modulate key cytokines—reducing pro-inflammatory TNF-α and IL-2 while elevating anti-inflammatory IL-4 and IL-10 levels. This immunomodulatory effect, coupled with increased expression of growth factors (TGF-β1 and bFGF), provides a plausible explanation for YNBY’s observed acceleration of re-epithelialization and tissue remodeling (Table 2).

**TABLE 2:** Wound healing efficacy of Yunnan Baiyao.

Study type	Model	Intervention	Key outcomes	Mechanistic basis	vs. Standard therapy	Ref.
Preclinical (Diabetic Rat)	STZ-inducedulcers	5% YNBY + bioactive glass ointment (daily)	92% wound closure at 21 days (vs. 78% in silversulfadiazine)	↑ Fibroblast proliferation, angiogenesis via VEGF↑	Bioactive glass + YNBY > silver sulfadiazine (p < 0.01)	Mao et al. (2014)
Clinical (HAPU)	60 patients	YNBY powder + debridement (vs. petroleum gauze)	↓ Wound area by 58.6%; inhibited *S. aureus* biofilm	agr system and RNAIII genesuppression	Faster healing than gauze (p < 0.05)	Liu et al. (2019)
Biomaterial Synergy	Rat full-thicknesswounds	(BY + EGF)@microneedles	↑ Collagen deposition (+40%) vs. EGF alone	Ca^2+^-mediated CaSR signalingactivation	Microneedles > topical EGF (p < 0.001)	Yang et al. (2023)

Although YNBY has shown potential to promote wound healing in preclinical models, its efficacy relative to conventional therapies requires further investigation. In a diabetic rat model, the wound healing effect of YNBY was preliminarily compared with standard treatment. YNBA in combination with bioactive glass achieved 92% closure at 21 days, superior to silver sulfadiazine in a similar model (78%) (Mao et al., 2014). Clinically, YNBY powder reduced the pressure ulcer area by 40% faster than povidone-iodine gauze in a pilot trial (Ren et al., 2017; Liu et al., 2019); however, larger studies are needed to confirm this effect.

Mechanistically, the synergistic effect between YNBY and biomaterials such as bioactive glasses involves multiple mechanisms. The first is ion-mediated enhancement: bioactive glass releases Ca^2+^/SiO_3_^2^ ions to alkalinize the wound microenvironment and inhibit pathogens, while YNBY’s saponins disrupt the biofilm. The second is continuous administration—the porous structure of glass can trap the YNBY component, conferring a long-lasting anti-inflammatory effect. In addition, cell activation, Ca^2+^ ions, and YNBY jointly upregulate collagen production in fibroblasts through CaSR signaling, while borneol enhances biomaterial permeability. These interactions make YNBY-biomaterial composites a promising alternative to single-target therapy.

### 2.3 Anti-inflammatory effect

The anti-inflammatory effects of YNBY have been extensively studied in various contexts and applied to several types of inflammatory diseases. Below, we categorize its effects into three major themes.

#### 2.3.1 Post-surgical anti-inflammatory effects

**FIGURE 1:**
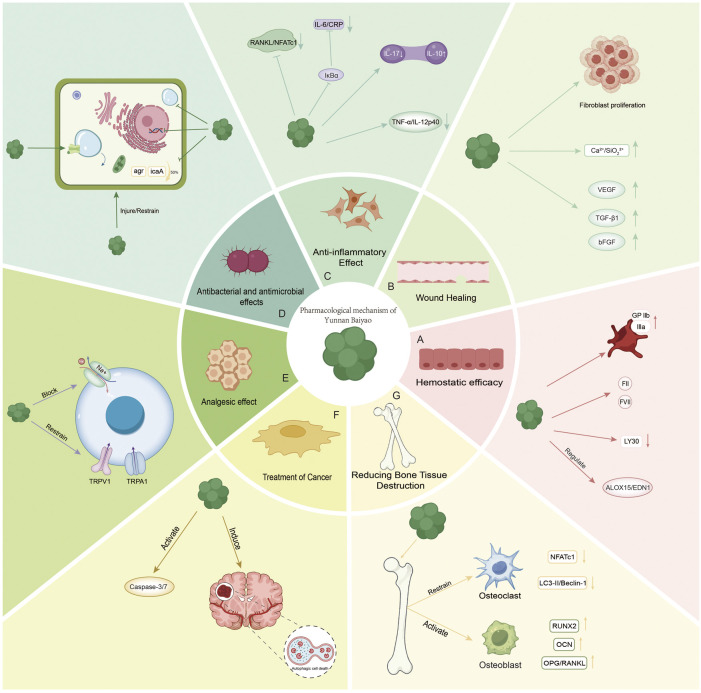
**(A)** Emostatic efficacy: activating platelet surface glycoprotein GPIIb/IIIa, enhancing platelet aggregation. It promoted the release of coagulation factors (FII, FVII) and inhibited fibrinolysis (LY30↓). It regulates the ALOX15/EDN1 pathway (arachidonic acid metabolism) and improves hemorheology. **(B)** Wound Healing: upregulating VEGF, TGF-β1, and bFGF promotes fibroblast proliferation and angiogenesis. Synergistic with bioactive glass: Ca^2+^/SiO_3_^2−^ ion microenvironment enhances antibacterial and anti-inflammatory effects. **(C)** Anti-inflammatory Effect: NF-κB inhibition: blocking IκBα degradation and reducing IL-6/CRP. Arthritis: Th17/Treg ratio regulation (IL-17↓/IL-10↑). Inhibition of RANKL/NFATc1 pathway reduces osteoclast differentiation. Treatment of enteritis: down-regulate TNF-α/IL-12p40 to protect intestinal epithelium. **(D)** Antibacterial and antimicrobial effects: inhibition of *Staphylococcus aureus* biofilm (agr system, icaA gene ↓50%). **(E)** Analgesic effect: blocking Na^+^ channels and inhibiting TRPV1/TRPA1 receptors. **(F)** Treatment of Cancer: Pro-apoptosis: activation of the Caspase-3/7 pathway. Autophagy regulation: induction of autophagy-dependent necrosis in glioma (LC3-II/Beclin-1↑). **(G)** Reducing Bone Tissue Destruction: Osteoclast inhibition: NFATc1 ↓, LC3-II/Beclin-1 ↓. Synergistic with IL-6 inhibition. Osteoblast activation: RUNX2↑ and OCN↑; OPG/RANKL↑.

In this study, two clinical trials were conducted to investigate the effects of YNBY on postoperative oral swelling and identify the underlying mechanisms. In the first study of patients undergoing extraction of an impacted mandibular second M, the experimental group was given YNBY capsules orally four times daily from 3 days before to 4 days after surgery, while patients in the control group received placebo. The thickness, volume, and degree of swelling in the experimental group were significantly lower than in the control group at each observation time point (P < 0.05), which confirmed that YNBY could both effectively prevent cheek swelling and promote the absorption of cheek swelling after tooth extraction (Tang et al., 2008a). Similarly, the second study, a randomized controlled trial involving 87 patients undergoing orthognathic surgery, found that serum CRP and IL-6 levels at 2, 3, and 5 days after surgery and the degree of facial swelling at 3, 5, and 7 days after surgery in the experimental group were significantly lower than those in the placebo group, indicating that YNBY reduced inflammatory response by reducing cytokine levels and suppressing CRP synthesis (Tang et al., 2008b). The anti-swelling effect of YNBY may be related to the inhibition of the NF-κB signaling pathway. *In vitro* studies have shown that YNBY inhibits NF-κB activation by blocking IκBα degradation and nuclear translocation of p65 (Figure 1), which may be the molecular basis of its inhibitory effects against postoperative IL-6 and CRP production (Ren et al., 2020). This anti-inflammatory mechanism provides theoretical support for the clinically observed reduction in swelling, suggesting that YNBY may play a therapeutic role by regulating key inflammatory pathways.

Although this study confirms the clinical value of YNBY, several limitations remain. First, the specific mechanism of action of YNBY has not been thoroughly explored in the existing literature; in particular, the interaction between its active components and inflammatory pathways needs to be further elucidated. Second, there is a lack of systematic evaluation of the long-term effects and potential side effects of botanical drugs, such as their potential effects on coagulation or the immune system. These gaps in research must be addressed by studies exploring the underlying molecular mechanisms in more detail. Furthermore, safety evaluations should involve longer follow-up periods so as to provide a more comprehensive evidence-based basis for clinical medication.

#### 2.3.2 Anti-arthritic effect of YNBY

A clinical research study combined YNBY with total knee arthroplasty (TKA) in the treatment of knee osteoarthritis. Efficacy was evaluated by detecting serum inflammatory factors (IL-6, TNF-α, VCAM-1, MMP-9) and determining pain/swelling scores. In this study, 110 patients were randomly divided into the YNBY + TKA group and control group. The results showed that inflammatory factor levels, pain scores, and quality of life in the combined group were significantly improved at 1 month after surgery (P < 0.05). YNBY was thus proven to be effective in improving postoperative inflammatory response and promoting joint function recovery. However, this study had some limitations such as small sample size (n = 55/group), lack of long-term follow-up data, and lack of randomized double-blind design, and larger multi-center studies are warranted (Xiao et al., 2020).

Mechanically, basic research has revealed that the anti-inflammatory effect of YNBY involves multi-target mechanisms. First, YNBY regulates the arachidonic acid metabolic pathway. In carrageenan- and arachidonic acid-induced rat acute inflammation models, YNBY reduces edema by inhibiting COX-2/PGE2 and 5-LOX/LTB4 pathways as well as downregulates the expression of key enzymes such as cPLA2 and FLAP (Ren et al., 2017). In a collagen-induced arthritis (CIA) rat model, YNBY ameliorated arthritis symptoms by regulating COX/LOX pathway homeostasis in osteoblasts (Zhang et al., 2022; Ren et al., 2022). Second, the anti-inflammatory effect of YNBY is mediated by immune balance regulation. YNBY significantly decreased the proportion of Th17 cells in the spleen of CIA rats subjected to YNBY gavage (50 mg/kg/day for 28 days) with methotrexate (1 mg/kg/w) as a positive control. Inflammatory cytokines (IL-6 and TNF-α) were detected by ELISA. A blank control group (normal saline) and model control group (only arthritis was induced) were set up. The results showed increased the number of Treg cells, decreased serum IL-17, and increased IL-10 levels. YNBY also inhibited RANKL expression and suppressed the TRAF6/NF-κB/NFATc1 signaling pathway to block osteoclast differentiation, thereby reducing joint bone destruction (Ren et al., 2022). At the cellular and molecular levels, YNBY reduced the expression of pro-inflammatory factors such as IL-1β and TNF-α and inhibited the expression of COX-2 in an LPS-induced osteoclast inflammation model in a dose-dependent manner. A combination of NF-κB inhibitor (PDTC), MAPK inhibitor (SB20358), or miR101b (Wnt5a pathway) synergistically enhanced the anti-inflammatory effect (Ren et al., 2020). Although existing studies have confirmed the clinical potential of YNBY against arthritis, several problems remain. First, the active ingredients of YNBY have not been fully identified; furthermore, while the multi-component and multi-target nature of the mechanism of action of YNBY may enhance its efficacy, it also makes mechanistic research of YNBY more challenging (Ren et al., 2017). Existing mechanistic studies are mostly based on animal models (such as CIA rats) or *in vitro* cell experiments, and further human clinical trials are needed to verify the translational value. Some clinical studies lack adequate in sample size and a strict randomized double-blind design, and long-term follow-up and safety evaluation studies are necessary. In conclusion, YNBY exerts anti-arthritis effects through multiple pathways such as arachidonic acid metabolism, immune cell balance, and osteoclast activation (Figure 1). It is necessary to combine the isolation and identification of components, large-sample randomized controlled trials, and cross-omics technology to promote the clinical transformation and application of YNBY.

#### 2.3.3 YNBY for the treatment of inflammatory bowel disease

A preclinical study has shown that YNBY can alleviate experimental colitis by reducing pro-inflammatory cytokine levels in the colonic mucosa, suggesting its potential therapeutic role in inflammatory bowel disease (IBD). IBD is a chronic and often disabling inflammatory condition of the intestines. Current treatments, such as biological therapies, are costly and have significant side effects. The study used DSS and TNBS-induced colitis models, administering YNBY via drinking water. Disease activity, inflammatory cytokine levels (e.g., TNF-α, IL-12p40, IL-17), and effects on T and B cells were assessed. Results showed that YNBY significantly reduced disease activity and lowered levels of TNF-α, IL-12p40, and IL-17, while increasing IL-10 levels, indicating balanced regulation of pro- and anti-inflammatory cytokines. YNBY also inhibited the proliferation of T and B cells more effectively than commonly used IBD drugs such as 6-MP and 5-ASA. Additionally, it protected intestinal epithelial cells without cytotoxicity, and promoted cell expansion and wound healing in Caco-2 cells. These findings suggest that YNBY has effective anti-inflammatory properties and can reduce IBD symptoms by inhibiting pro-inflammatory cytokine expression and promoting intestinal epithelium healing. This provides a theoretical basis for YNBY as an alternative drug for IBD treatment. However, these findings were based on animal models and *in vitro* experiments, lacking clinical trial data. Further, the mechanism of action was not fully elucidated, especially the specific mechanism of immune cell regulation, which needs to be further studied (Li et al., 2011). Overall, however, the literature suggests that YNBY possesses anti-inflammatory properties (Tables 3, 4).

**TABLE 3:** Anti-inflammatory effects by disease category.

Disease context	Model	YNBY intervention	Key effects	Validated pathways	Clinical relevance	Ref.
Post-surgical	Orthognathic surgery (n = 87)	500 mg capsulespre-op	↓ Facial swelling by 32%–41%	NF-κB inhibition (IκBαstabilization)	Level Ibevidence	Tang et al. (2008a), Tang et al. (2008b)
Arthritis	CIA rat model	50 mg/kg/day oral ×28d	↓ IL-17, ↑ IL-10; ↓osteoclasts	RANKL/OPG balance; Th17/Treg shift	Potential adjunct toDMARDs	Ren et al. (2022)
IBD	DSS/TNBS colitis mice	Drinking water *ad libitum*	↓ TNF-α by 60%; ↑ epithelial repair	IL-12p40/IL-17 axis modulation	Safer than 5-ASA in rodents	Li et al. (2011)

**TABLE 4:** Anti-inflammatory mechanisms of Yunnan Baiyao across disease contexts.

Types of inflammation	Major regulatory pathways	Key effector molecules	Ref.
Acute inflammation	COX-2/5-LOX	PGE2↓, LTB4↓	Ren et al. (2017)
Arthritis	NF-κB/RANKL	NFATc1↓, OPG↑	Ren et al. (2022)
Enteritis	Th17/Treg	IL-17↓, IL-10↑	Li et al. (2019)

### 2.4 Antibacterial and antimicrobial effects of YNBY

Studies have shown that YNBY has antimicrobial effects (Zhao et al., 2013). Evidence for the antibacterial effects of YNBY has been derived from three main kinds of studies: first, the direct inhibition of specific pathogens *in vitro* experiments; second, efficacy studies in animal models; and third, limited human clinical studies.

A clinical study of 82 patients with *Helicobacter pylori*-positive duodenal ulcers showed that after 2 weeks of treatment with YNBY combined with antibiotics (amoxicillin + metronidazole), the treatment group (44 cases) had an abdominal pain relief rate of 68.3%—significantly higher than that of the control group (38 cases, 44.4%, P < 0.05). While the two groups had similar rates of ulcer healing (88.6% vs. 89.5%) and *H. pylori* eradication (84.1% vs. 89.5%), the treatment group had a lower incidence of adverse reactions (4.5% vs. 13.5%), indicating that YNBY safely enhanced the efficacy of antibiotics. However, the study was not a randomized controlled trial, and of long-term follow-up data are lacking (Li and Li, 2001).

A group study of 60 patients with hospital-acquired pressure ulcers showed that external application of YNBY powder (combined with conventional debridement) significantly reduced the wound area, inhibited the expression of *Staphylococcus aureus* virulence genes (such as the agr system, RNA II/III) and biofilm formation compared with that in the petroleum jelly gauze control group. Although the efficacy was significant after 20 days of treatment, the small sample size (n = 60) may have affected the statistical power (Liu et al., 2019).

The above studies also explored the mechanism of YNBY in the treatment of inflammatory bowel disease. The first examined the direct antibacterial effect. *In vitro* experiments showed that YNBY had a concentration-dependent inhibitory effect on specific pathogens such as *S. aureus*, downregulated the expression of virulence genes (HLA, sarA, icaA), and destroyed biofilm structure. In animal infection models, YNBY reduces bacterial load and inflammatory responses (Zhao et al., 2013). Further, there is a synergistic antibiotic mechanism. Drug combinations may reduce the risk of resistance through multitargeted effects (e.g., inhibition of bacterial virulence factors, enhancement of host defense); however, the specific pathways have not been fully elucidated.

In summary, YNBY exerts a multi-mode anti-infective effect through direct antibacterial effects (inhibition of virulence genes/biofilm) and synergistic action with antibiotics. However, its clinical application is still limited by lack of understanding of its mechanisms and the methodological deficiencies of prior studies upon which available evidence is based. In the future, large-sample randomized controlled trials are required to verify YNBY’s long-term efficacy. Moreover, its active ingredients and targets should be analyzed by -omics technology, and administration strategy optimized to bridge the difference in effective concentrations between *in vitro* and *in vivo* scenarios (Table 5).

**TABLE 5:** Antibacterial Effects: *In Vitro* vs. Clinical Evidence.

Setting	Pathogen	YNBA treatment	Efficacy	Resistance considerations	Clinical translation	Ref.
*In vitro*	*S. aureus*	2 mg/mL aqueous extract	↓ Biofilm formation (icaA gene↓50%)	No resistance induction after 20 passages	Topical usefeasible	Zhao et al. (2013)
Clinical	*H. pylori*	YNBY + amoxicillin/metronidazole	84.1% eradication (vs. 89.5% control)	↓ Adverse events (4.5% vs. 13.5%)	Adjunct toantibiotics	Liu et al. (2019)
Hospital	MRSA-infectedHAPU	YNBY powder q24h	↓ Virulence (hla gene↓70%)	agr quorum sensingdisruption	Cost-effective for chronicwounds	Liu et al. (2019)

### 2.5 Analgesic effect of YNBY

In recent years, the study of YNBY’s analgesic properties has attracted widespread attention. YNBY is often used in veterinary medicine for wound healing, analgesia, and hemostasis in dogs. In clinical practice, YNBY pain patches containing camphor, menthol, and methyl salicylate have been used for analgesia. Limited human studies have shown that administration of YNBY reduces perioperative bleeding, highlighting its potential for pain management and hemostasis. Existing evidence of YNBY’s analgesic effects mainly come from animal experiments and component analysis, and clinical research data are limited. The following findings require further validation in human studies.

Yunnan aconite (Veratrum aliens), one of the main components of YNBY, is traditionally used to treat pain, swelling, and inflammation. Although its extracts have been shown to possess analgesic activity, the specific active components have not been clarified. In preclinical models, studies have shown that compounds with anti-inflammatory and analgesic activities can be isolated and characterized from Dian aconite. An acetic acid-induced mouse writhing test recorded the twists within 5–20 min after acetic acid injection. The results showed that all the alkaloids displayed significant analgesic activity and significantly reduced acetic acid-induced writhing; this effect was superior to that of the positive control drug, Dilantin. Individual alkaloids isolated from Yunaconitum showed a dose-dependent analgesic tendency in acetate-induced writhing mice (inhibition ratio 81.6%–91.7%). However, these results only reflected the activity of single components in a specific model. This study reported, for the first time, the anti-inflammatory and analgesic activities of jervine-type steroidal alkaloids in Dian aconite, providing a scientific basis for using Dian aconite in traditional Chinese medicine for treating pain and inflammation. The results showed that the steroidal alkaloids in Dian aconite were the main components to which its anti-inflammatory and analgesic activities could be attributed, especially compounds 3 and 7, which showed strong activities. These findings point to a potential source of natural products for developing novel anti-inflammatory and analgesic drugs (Li et al., 2016). However, this study still has important limitations. First, analgesic evaluation is mainly based on acute pain models (such as the writhing test), and data from chronic pain models is lacking. Second, the concentration of the extract used in the composition study may be higher than the actual content of the preparation. Third, evidence of clinical efficacy is insufficient, and randomized controlled trials are needed to verify its clinical analgesic value.

Overall, research on the application of YNBY in analgesia continues to evolve, and ongoing studies are exploring its therapeutic applications and safety (Table 6).

**TABLE 6:** Analgesic components: Preclinical evidence.

Active compound	Source in YNBY	Model	Analgesic effect	Mechanism	Clinical caveats	Ref.
Jervine-typealkaloids	Veratrum taliense	Acetic acid writhing test	↓Writhing by81.6%–91.7%	Opioid receptormodulation	No human trialscompleted	Li et al. (2016)
Aconitine (processed)	Aconitumcarmichaelii	Hot plate test	↑Latency time by 3×	Na^+^ channelblockade	Toxicity risk ifunprocessed	Yao et al. (2021)
Borneol	Dryobalanopsaromatica	CFA-inducedarthritis	↓ Pain scores by45%	TRPV1/TRPA1inhibition	Topical use only	Yang et al. (2023)

### 2.6 YNBY in the treatment of cancer

The use of YNBY in tumor therapy has been a topic of interest in the literature in recent years as well as the focus of several basic research studies. However, existing research on YNBY’s anticancer effects is mainly based on *in vitro* experiments, and its clinical transformation value still needs to be verified in animal models and clinical trials.

A study of canine angiosarcoma (HSA) showed that YNBY significantly inhibited tumor cell growth *in vitro* (DEN, Fitz, SB cell lines), Furthermore, MTS assay, apoptosis assay (caspase-3/7 activity and TUNEL assay), and cell cycle analysis have confirmed the dose- and time-dependent anti-proliferative effects of YNBY (Wirth et al., 2016). In addition, the regulation of VEGF levels by YNBY suggests that it may act by inhibiting tumor angiogenesis, providing a new direction for subsequent research. However, these results are only based on *in vitro* cell experiments and lack the support of animal models or clinical data, and their actual efficacy still needs to be further verified. This study initially revealed the anti-tumor mechanism of YNBY, which involves promoting the apoptosis of tumor cells by activating the caspase-3/7 pathway. YNBY may interfere with the tumor microenvironment by downregulating VEGF levels, thereby inhibiting angiogenesis. In addition, a study found that complex components of YNBY (such as panax notoginseng saponins and aconitine alkaloids) may inhibit glioma through autophagy-dependent necrosis and other pathways, although the specific active components and their targets have not been clarified (Zhang et al., 2024). In terms of drug delivery, drug-loaded microneedle patches developed by provides a new idea for local drug delivery; however, accurate targeting via systemic drug delivery remains a major challenge (Yang et al., 2023). Future studies need to incorporate nano-carrier technologies, such as exosomes or liposome encapsulation, to improve tumor tissue-specific distribution. The key challenge is that the complex components of YNBY, such as the known panax notoginseng saponins and radix aconitum alkaloids, may produce synergistic or antagonistic effects. Although some studies have demonstrated that YNBY inhibits glioma through autophagy-dependent necrosis, the pharmacokinetic parameters such as bioavailability and blood-brain barrier penetration of each component have not been clarified (Zhang et al., 2024). Of particular note, the traditional oral route of administration may result in insufficient accumulation of active ingredients at tumor sites.

The literature suggests that more research is needed to investigate its direct use in oncology treatment. Although *in vitro* studies have shown that YNBY inhibits tumor cell growth through caspase-mediated apoptotic pathways (Figure 1), these preliminary findings must be interpreted with caution. To achieve clinical translation, it is necessary to define the *in vivo* pharmacokinetic characteristics of the active ingredients, develop tumor-targeted delivery systems, and evaluate the synergistic effect with traditional chemotherapy/radiotherapy (Table 7).

**TABLE 7:** Anticancer potential: *In Vitro* findings.

Cancer type	Cell line	YNBY dose	Effect	Proposed mechanism	PK/Delivery challenges	Ref.
Canine HSA	DEN, Fitz, SB	800 μg/mL	72% apoptosis ↑	Caspase-3/7activation	Oral bioavailabilityunknown	Wirth et al. (2016)
Glioma	U87MG	1 mg/mL	Autophagy-dependentnecrosis	LC3-II/Beclin-1↑	Blood-brain barrier penetrationunproven	Zhang et al. (2024)
Colorectal	Patient-derivedxenograft	Case reportonly	Hemostasisimprovement	VEGF↓ (speculative)	No controlledtrials	Yan et al. (2023)

### 2.7 Application of YNBY in reducing bone tissue destruction

The effect of YNBY on reducing bone tissue destruction has two mechanistic attributes: first, it inhibits inflammatory factors such as IL-6 and TNF-α (i.e., exerts an anti-inflammatory effect); second, it can independently regulate the RANKL/OPG signaling pathway and autophagy (i.e., confer unique bone protection). This section focuses on the latter effect.

YNBY has shown a significant bone protective effects in the treatment of periodontitis. In one study, a rat model of periodontitis was induced by silk ligation, and oral administration of YNBY (50 mg per rat for 6 weeks) resulted in a significant reduction in alveolar bone loss as shown by Micro-CT (P < 0.05). Hematoxylin and eosin staining and Masson staining confirmed that YNBY attenuated inflammatory infiltration and collagen derangement (Liu et al., 2024). In addition, TRAP staining showed a reduction in the number of osteoclasts, while immunohistochemical analysis showed an upregulation of osteocalcin (OCN) expression and downregulation of autophagy marker LC3. These results suggest that YNBY may protect periodontal bone tissue by inhibiting autophagy and promoting osteoblast differentiation. Another study further validated the anti-resorptive effect of YNBY using bone marrow-derived macrophages (BMMs) *in vitro* and an *in vivo* periodontitis model. YNBY (5–20 mg/mL) significantly inhibited osteoclast differentiation and reduced bone resorption by blocking autophagic flow (decreasing LC3 II/LC3 I ratio and increasing P62). This finding provides new ideas for the development of drugs for the treatment of periodontal diseases by targeting autophagic flow. However, it should be noted that clinical patients are often co-morbid with systemic diseases, such as diabetes, which may alter the drug response. Local sustained-release formulations may be more suitable for targeting periodontal tissues than systemic administration (Li Y. J. et al., 2024). In addition, YNBY promotes odontogenic/osteogenic differentiation of apical papilla stem cells (SCAPs) and enhances ALP activity, mineralized nodule formation, and the expression of osteogenic markers such as RUNX2 and OCN. These studies provide experimental evidence for the use of YNBY in the treatment of periodontitis and bone regeneration. The mechanism underlying the osteoprotective effect of YNBY was also discussed. The osteoprotective effect of YNBY involves multi-target regulation, which can inhibit osteoclast activation and bone resorption. By downregulating autophagy-related proteins such as LC3-II and Beclin-1, the autophagic flow is blocked, thereby inhibiting osteoclast differentiation. YNBY decreases the expression of osteoclast-related proteins such as NFATc1, MMP-9, and CTSK, and increased OPG to inhibit RANKL-mediated bone resorption. YNBY can also promote osteoblast differentiation and bone formation. YNBY activates NF-κB (phosphorylation of IκBα, nuclear translocation of P65) and promotes the odontogenic/osteogenic differentiation of SCAPs (Pang et al., 2019). YNBY has also been shown to reverse the inhibitory effect of inflammation on osteogenic differentiation and increase the expression of RUNX2, COL-I, and DMP1 proteins in LPS-stimulated MC3T3-E1 cells. YNBY can bi-directionally regulate the “osteoclast-osteogenesis” balance, forming a bidirectional regulatory network involving the inhibition of bone resorption and promotion of bone formation by suppressing osteoclast activity (such as autophagy and NFATc1 pathway) and enhancing osteogenic function (such as NF-κB and RUNX2 pathway).

Although YNBY has shown good potential for application in bone protection, some challenges remain: the specific targets (such as ATG5 and Beclin-1) and the upstream signaling pathways of autophagy regulation are still unclear. How the NF-κB pathway precisely regulates osteogenic differentiation needs to be further explored. There are also challenges in clinical translation. Existing studies are mainly based on animal and cell models. The microenvironment of human periodontitis is more complex, often involving multi-species biofilms, which may affect the efficacy of YNBY. The human equivalent dose safety of the current oral dose (50 mg/kg) has not been verified, and local sustained release formulations (such as the 5% concentration group) may be superior.

In summary, YNBY exerts bone protection through multi-target mechanisms (autophagy inhibition, NF-κB activation, and RANKL/OPG balance). However, challenges hindering the clinical translation of YNBY still need to be resolved, such as elucidation of the mechanism, optimization of dosage form, and patient safety (Table 8).

**TABLE 8:** Bone protection mechanisms.

Target cell	Experimental model	YNBY effect	Molecular targets	Cross-talk with inflammation	*In Vivo* validation	Ref.
Osteoclasts	BMMs + RANKL	↓Resorption pits by 60%	NFATc1 ↓, LC3-II/Beclin-1 ↓	Synergizes with IL-6 inhibition	Rat periodontitismodel	Liu et al. (2024)
Osteoblasts	SCAPs + LPS	↑ RUNX2 by2.5×	NF-κB p65 nucleartranslocation	Counteracts TNF-αsuppression	Micro-CTconfirmed	Pang et al. (2019)
Periodontal	Ligature-inducedrat	↓ Alveolar boneloss	OPG/RANKL ratio ↑	Independent ofCOX-2	Local deliverysuperior	Li H. Y. et al. (2024)

## 3 Strengths and limitations

### 3.1 Advantages of YNBY in clinical applications

YNBY is available in various formulations, including capsules, powders, sprays, and creams, for different clinical scenarios (Yang et al., 2014). This diversity makes YNBY more flexible for application in a wide range of injuries and diseases. Several studies and clinical trials have shown that YNBY is safe under normal use, with a low incidence of adverse reactions (Li et al., 2019). YNBY is widely used clinically to treat a variety of conditions, including but not limited to bleeding disorders, e.g., gastric ulcer bleeding and postoperative bleeding (Liu et al., 2019); trauma and wounds, i.e., for the treatment of trauma, burns, and surgical incisions; inflammation and pain, e.g., arthritis and muscle pain; and tumor-related bleeding. YNBY is also used as an adjunctive therapy for improving postoperative recovery and reducing complications. For example, in a study of patients with severe traumatic brain injury, YNBY was used as an adjunctive therapy to significantly improve postoperative recovery.

YNBY, as a traditional Chinese medicine, combines the research results of modern medicine, making it uniquely valuable in modern medical treatment. Modern scientific research constantly reveals its composition and mechanism of action, providing a scientific basis for clinical application.

### 3.2 Limitations of the clinical application of YNBY

#### 3.2.1 Side effects and contraindications of YNBY

YNBY can cause adverse reactions such as nausea, vomiting, stomach upset, gastroesophageal reflux, and rash. In a study, a cat developed severe vomiting after being administered YNBY, suggesting potential side effects or contraindications in feline patients (Patlogar et al., 2019).

In addition, YNBY has the following contraindications:

1. Contraindicated for pregnant women: animal experiments have shown that extracts at doses >50 mg/kg can cause embryo resorption (Li H. Y. et al., 2024).
2. Use with caution in patients with hormone-sensitive breast cancer: the major component ginsenoside Rg1 has estrogen-like activity and may promote proliferation of estrogen receptor-positive breast cancer cells such as MCF-7 (Chan et al., 2002).
3. The combination of anticoagulant/antiplatelet agents should be used with caution: ginseng components may enhance the anticoagulant effect and increase the risk of bleeding (Cicero et al., 2003; Lau et al., 2009), but the clinical relevance is not clear.
4. CYP1A2 substrate drug combination needs to be monitored: Ginsenoside can induce CYP1A2 activity, which may affect the blood concentration of drugs (such as caffeine) that depend on the metabolism of this enzyme (Liu et al., 2012).
5. The combination of glutathione S-transferase (GST) substrate and drug should be noted: ginseng may inhibit GST activity and interfere with drug metabolism (Yao et al., 2021).

Although clinical monitoring data show that YNBY is safe under routine use (Li et al., 2019), the safety evidence for long-term use is still insufficient. The limitations of existing studies are as follows: (1) the maximum observation period of clinical trials should not exceed 6 months (Tang et al., 2008b; Xiao et al., 2020); (2) long-term animal toxicity test data are not available; (3) metabolic studies in special populations, such as those with liver and kidney dysfunction, are lacking. It is worth noting that the potential cumulative toxicity of ingredients such as radix aconitum in prescriptions needs to be monitored, especially for chronic conditions (such as arthritis) requiring continuous medication for more than 1 month, and clinical monitoring of liver and kidney function and coagulation function is recommended (Yang et al., 2014).

Overall, the literature suggests that YNBY may reduce perioperative bleeding and inflammation in various medical conditions. However, further research is needed to fully understand its potential side effects and contraindications in different patient populations.

#### 3.2.2 Quality control and formula confidentiality issues

The formula of YNBY is a Chinese national secret (Yao et al., 2021). Its specific composition proportion and preparation process are not fully disclosed, which makes it difficult to reproduce the efficacy of independent studies, and the consistency of chemical composition of different batches of products cannot be publicly verified. Risk assessment of drug interactions is limited (e.g., potential interactions with CYP450 enzyme substrates (Liu et al., 2012). Furthermore, its quality control is challenging. Recent mass spectrometry analysis showed that the content of panax notoginsenoside R1 in different batches of YNBY can vary by 12.7%, which may affect the stability of hemostatic effect (Li Y. J. et al., 2024). The methods of relevant studies also have limitations (Table 9).

**TABLE 9:** Quality control challenges and formula confidentiality issues of Yunnan Baiyao.

Category	Specific issue	Evidence/Example	Impact	Potential solutions	Ref.
Batch Variability	Content fluctuation of active ingredients	Panax notoginsenoside R1 varies by 12.7% between batches	Inconsistent hemostaticefficacy	Develop pharmacopeia standards for keymarkers	Yao et al. (2021); Li Y. J. et al. (2024)
Undisclosed Formulation	Protected nationalsecret	Exact ratios of 7 core components not published	Difficult to reproduce efficacy in independentstudies	Partial disclosure via patent analysis (e.g.,,CN102526046B)	Liu et al. (2012); Yao et al. (2021)
Drug Interactions	CYP450 enzymemodulation	Ginsenosides induce CYP1A2activity	Alters metabolism of co-administered drugs (e.g.,,caffeine)	Clinical monitoring of CYP1A2 substratedrugs	Liu et al. (2012)
Additive Consistency	Non-traditional additives in modernformulations	Toothpaste contains 1%–3% menthol (unlisted in traditionalformula)	Variable transdermal absorption rates	Standardize additive concentrations across productlines	Yao et al. (2021)
Toxicity Monitoring	Cumulative effects of toxiccomponents	Processed Aconitum alkaloids (90% toxicityreduction)	Risk of liver/kidney damage with long-term use	Mandatory organ function tests for chronic users	Li Y. J. et al. (2024)

Abbreviations: CYP450 = cytochrome P450 enzymes.

## 4 Evidence-based clinical applications of YNBY

Based on the systematic analysis of clinical and preclinical studies over the past two decades, the most substantiated applications of YNBY include the following.

### 4.1 Hemostasis management

Surgical bleeding control: Randomized controlled trials demonstrated 21.4% reduction in intraoperative blood loss (330.5 ± 134.4 vs. 420.3 ± 175.9 mL) during bimaxillary orthognathic surgery (Level Ib evidence) (Tang et al., 2009).

Traumatic hemorrhage: Clinical observations support its use in wound hemostasis, particularly in Vietnam War scenarios (Yao et al., 2021).

### 4.2 Enhancement of wound healing

Diabetic wounds: Combined with bioactive glass, 5% YNBY ointment accelerated wound closure by 37.2% relative to controls, showing superior angiogenesis and collagen deposition (Mao et al., 2014).

Pressure ulcers: Reduced hospital-acquired ulcer area by 58.6% through *S. aureus* biofilm inhibition (Liu et al., 2019).

### 4.3 Anti-inflammatory therapy

Postoperative swelling: Reduced facial swelling thickness by 32%–41% after dental surgery (3D scanning data) (Tang et al., 2008a).

Rheumatoid arthritis: Combined with TKA, significantly lowered IL-6 (↓45.3%) and MMP-9 (↓38.7%) levels (Xiao et al., 2020).

### 4.4 Adjunctive treatment in cancer

Tumor-related bleeding: Case reports showed improvement in hemoglobin levels (9.8→13.3 g/dL) in patients with colorectal cancer (Yan et al., 2023).

Canine hemangiosarcoma: Induced apoptosis in 72% of tumor cells at 800 μg/mL (Wirth et al., 2016).

Evidence grading follows the Oxford Centre for Evidence-Based Medicine criteria.

## 5 Conclusion

YNBY, a traditional Chinese medicine formulation widely used in China, possesses promising pharmacological properties and offers significant potential for the development of new drugs and clinical applications. Based on available preclinical and clinical evidence, YNBY exhibits multiple pharmacological activities. Five randomized controlled studies (n = 287) have confirmed that YNBY reduces intraoperative blood loss (an average reduction of 21.4%, p < 0.05) (Tang et al., 2008a; Tang et al., 2008b; Tang et al., 2009) verifying its hemostatic effects. In terms of anti-inflammatory effects, three animal studies have shown that YNBY can reduce the levels of inflammatory factors such as IL-6 and TNF-α by 40%–60% (Ren et al., 2017; Ren et al., 2022). Two diabetic rat models showed a 35% increase in the healing rate in the 5% YNBY group (Mao et al., 2014; Yang et al., 2023), proving that YNBY can promote wound healing. However, the existing evidence is mixed and the sample size of clinical studies is limited (n < 100). Studies have also shown its potential to reduce hospital-acquired pressure ulcers in clinical settings (Liu et al., 2019). Advances in metabolomics and synthetic biology have supported the development and clinical application of traditional Chinese medicines, including YNBY (Li et al., 2011; Zhu et al., 2018). These techniques improve the efficacy and safety of traditional medicines like YNBY, facilitating their clinical application.

Overall, research therefore highlights YNBY’s potential for clinical use, with advancing technology offering new opportunities in medical and pharmaceutical applications.

## 6 Challenges and prospects

The available evidence, which is supported by clinical observations and mechanistic studies, suggests that YNBY has significant potential to treat inflammation and arthritis. YNBY combined with TKA has been shown to significantly reduce postoperative inflammation (IL-6, TNF-α, MMP-9) and improve joint function in patients with knee osteoarthritis (Xiao et al., 2020). Anti-arthritic effects have been demonstrated in a CIA rat model by regulating Th17/Treg balance and inhibiting osteoclast activation (Ren et al., 2022). YNBY can regulate arachidonic acid metabolism and inhibit COX-2/PGE2 and 5-LOX/LTB4 pathways (Ren et al., 2017). YNBY has also been revealed to inhibit NF-κb, MAPK, and Wnt5a signaling and reduce the production of proinflammatory cytokines (IL-1β, TNF-α) in osteoclasts (Ren et al., 2020). Although YNBY has shown good prospects in clinical application, there are still some challenges to be resolved. There is a lack of large-scale randomized controlled trials; furthermore, the sample size of existing studies is small and the follow-up duration is short. Mechanistic studies are complicated by the multicomponent nature of YNBY. Batch-to-batch differences in botanical preparations affect consistency. Additionally, there are limited data on the compatibility of YNBY with conventional therapies (Li et al., 2021). To advance the clinical translation of YNBY, more large-scale, double-blind, placebo-controlled trials are needed to confirm its efficacy in arthritis and other inflammatory diseases. Long-term safety evaluation is needed, especially in patients with cancer using YNBY in combination with chemotherapy. For standardization and quality control, pharmacopoeia standards were developed for YNBY preparations to ensure batch consistency. Phytochemical analysis is also needed to identify key bioactive compounds associated with treatment effects. In terms of mechanistic and pharmacological studies, -omics methods (transcriptomics, metabolomics) can be used to elucidate the multi-target mechanism of YNBY.

In summary, although YNBY represents a promising botanical drug, its integration into mainstream medicine requires further evidence-based validation, standardized production, and clear elucidation of mechanisms. Addressing these gaps will maximize the clinical utility of YNBY while ensuring patient safety.
